# Distinctive expression patterns of hypoxia-inducible factor-1α and endothelial nitric oxide synthase following hypergravity exposure

**DOI:** 10.18632/oncotarget.9372

**Published:** 2016-05-14

**Authors:** Gun Yoon, Choong Sik Oh, Hyun-Soo Kim

**Affiliations:** ^1^ Department of Obstetrics and Gynecology, Pusan National University Yangsan Hospital, Pusan National University School of Medicine, Yangsan-si, Gyeongsangnam-do, Republic of Korea; ^2^ Aerospace Medicine Research Center, Republic of Korea Air Force Aerospace Medical Center, Cheongju-si, Chungcheongbuk-do, Republic of Korea; ^3^ Department of Pathology, Severance Hospital, Yonsei University College of Medicine, Seoul, Republic of Korea

**Keywords:** endothelial nitric oxide synthase, hypoxia-inducible factor-1α, hypergravity, heart, liver, Pathology Section

## Abstract

This study was designed to examine the expression of hypoxia-inducible factor-1α (HIF-1α) and the level and activity of endothelial nitric oxide synthase (eNOS) in the hearts and livers of mice exposed to hypergravity. Hypergravity-induced hypoxia and the subsequent post-exposure reoxygenation significantly increased cardiac HIF-1α levels. Furthermore, the levels and activity of cardiac eNOS also showed significant increase immediately following hypergravity exposure and during the reoxygenation period. In contrast, the expression of phosphorylated Akt (p-Akt) and phosphorylated extracellular signal-regulated kinase (p-ERK) showed significant elevation only during the reoxygenation period. These data raise the possibility that the increase in cardiac HIF-1α expression induced by reoxygenation involves a cascade of signaling events, including activation of the Akt and ERK pathways. In the liver, HIF-1α expression was significantly increased immediately after hypergravity exposure, indicating that hypergravity exposure to causes hepatocellular hypoxia. The hypergravity-exposed livers showed significantly higher eNOS immunoreactivity than did those of control mice. Consistent with these results, significant increases in eNOS activity and nitrate/nitrite levels were also observed. These findings suggest that hypergravity-induced hypoxia plays a significant role in the upregulation of hepatic eNOS.

## INTRODUCTION

Hypoxia can stimulate the expression of numerous angiogenic factors *via* induction of hypoxia-inducible factor-1 (HIF-1), a heterodimeric protein composed of HIF-1α and HIF-1β subunits. Hypoxia results in the translocation of HIF-1α to the nucleus, where it binds to HIF-1β to form an active complex that initiates the transcription of angiogenic factors. While HIF-1α expression tightly correlates with cellular oxygen levels, it can also be increased or decreased by a variety of factors, including cytokines, signaling pathways, genetic alterations, and environmental factors. In addition, although the role of HIF-1α during hypoxia has been well established, the upstream signaling events that stimulate HIF-1α expression in response to reoxygenation are yet to be characterized.

Hypoxia and reoxygenation activate MEK/ERK and PI3K/Akt signaling [1]. In addition, activated ERK and Akt pathways have been identified as potent modulators of HIF-1α expression [2-6]. Mitogen-activated protein kinases (MAPKs) are serine/threonine kinases that activates or suppresses a variety of cellular functions, including proliferation, differentiation, and apoptosis [7, 8]. The extracellular signal-regulated kinase (ERK) pathway, which is activated by MAPK/ERK kinase (MEK), mediates a number of cellular fates, including growth, proliferation, and survival [9, 10]. The serine/threonine kinase Akt also influences these important cellular events and is activated by phosphoinositide 3-kinase (PI3K)-dependent signaling pathways [11, 12].

Nitric oxide (NO) influences myocardial function during physiological and pathological states [13-17]. In fact, its role in cardiac hypoxia has become one of the most widely investigated topics in basic cardiovascular research in recent years. Studies investigating the role of NO in cardiac function often report contradictory effects (i.e., NO signaling can be harmful or protective) [16]. NO is synthesized by NO synthase (NOS), a family of isoenzymes with characteristic functional and regulatory properties. In the vasculature, NO is produced mostly by endothelial NOS (eNOS), which is involved in physiological endothelial function and cardiovascular homeostasis. Cardiac endothelial cells and ventricular cardiomyocytes express eNOS [18-20]. NO production in the endothelium is increased or decreased by modulating eNOS expression and activity. Several compounds and pathophysiological conditions that stimulate or inhibit eNOS gene expression have been described. [21]. Accumulating evidence suggests that hypoxia results in increased cardiac eNOS expression and NO production [22, 23]. Some studies have also shown that hypoxic ventricular cardiomyocytes produce more intracellular NO than normoxic control cells [24-26].

Immunohistochemical studies with antibodies against eNOS have localized the enzyme in various cell types in many tissues, including the liver [27]. Several experimental models of altered hepatic eNOS expression have emphasized the crucial role of this enzyme in diverse pathophysiological conditions of the liver. For example, lipopolysaccharide or lipoteichoic acid treatment of an *in vivo* sepsis model significantly increased eNOS mRNA expression [28]. Another study using a model of alcoholic liver injury revealed alcohol-related attenuation of eNOS activity in rats fed an ethanol-containing liquid diet [29]. Furthermore, rats with carbon tetrachloride-induced cirrhosis exhibit significantly lower hepatic eNOS activity than do control animals [30]. eNOS plays a role in microcirculatory and immunomodulatory responses during hepatic hypoxia/reoxygenation injury. In a mouse orthotopic liver transplantation experiment, eNOS-knockout mice exhibited more severe microcirculatory disturbances and hepatocellular necrosis following hepatic hypoxia/reoxygenation than their wild-type counterparts [31].

The development of a new generation of combat aircrafts with extended flight capabilities has raised the problem of crew protection against various internal organ diseases due to sustained exposure to high gravitational (G) force (+Gz). Cardiovascular function is the primary pathophysiological target of G forces, which act in the same direction as the vascular axis of the sitting pilot. G force is a unique stress that principally results in impaired coronary blood flow when the inertial vector is in the head-foot direction. Previous studies have shown that exposure to hypergravity produces marked changes in cardiac performance characterized by arrhythmias and electrocardiographic abnormalities which probably occur as a result of cardiac hypoxia [32, 33]. Changes in heart position, distribution of central blood volume, and disorders in heart chamber filling decrease the stroke volume and consequently reduce cardiac output despite tachycardia. This reduced cardiac output decreases coronary blood flow and results in hypoxic insult to the heart. Furthermore, exposure to +Gz severely decreases blood flow to the abdominal organs, including the spleen, pancreas, kidneys and liver—an apparent effort to maintain blood flow to the brain and heart [34, 35]. It is likely that the changes in visceral blood flow are the result of some combination of hypergravity-induced cardiovascular reflex response and emotional stress.

The effects of exposure to hypergravity on cardiovascular function have been the subject of numerous studies. However, the field is lacking crucial information regarding potential changes in HIF-1α, ERK, Akt, and eNOS expression in heart tissue following hypergravity exposure. Moreover, no information is available on alterations in hepatic eNOS expression and activity following hypergravity exposure. This study was designed to investigate whether hypergravity exposure alters cardiac HIF-1α levels and/or hepatic eNOS expression and activity and activates the ERK and/or Akt pathways.

## RESULTS

### HIF-1α expression in hypergravity-exposed hearts

**Figure 1:**
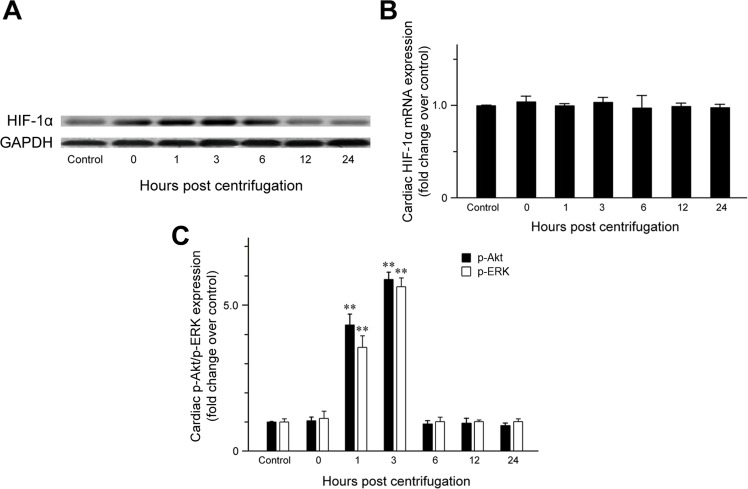
Effect of exposure to hypergravity on cardiac expression of HIF-1α, p-Akt, and p-ERK **A.** Western blotting detected HIF-1α as a 120-kDa band, the levels of which increased immediately after centrifugation. This increase persisted until 6 hr post-centrifugation. Afterwards, protein levels returned to basal levels. **B.** Quantitative RT-PCR analysis revealed no significant alterations in mRNA levels of HIF-1α during hypoxia or reoxygenation. **C.** In contrast to HIF-1α and eNOS, elevated p-Akt and p-ERK expression was observed during the reoxygenation period (1-3 hr), but not immediately after hypoxia (0 hr). **P < 0.01.

The expression of cardiac HIF-1α was examined to confirm that hypergravity induces hypoxia in the hearts of centrifuged mice. HIF-1α expression was significantly elevated immediately after centrifugation (Figure 1A), indicating that hypergravity exposure induces cardiac hypoxia. Unexpectedly, HIF-1α levels further increased from 1 to 3 hr post-exposure. After a slight decrease at 6 hr post-exposure, HIF-1α levels decreased further to the level in the control group. We next analyzed HIF-1α mRNA levels to determine whether the changes in HIF-1α levels were the result of increased transcription or protein stability. No significant changes in HIF-1α mRNA were observed, indicating that hypergravity exposure altered HIF-1α levels independent of transcription of the HIF-1α gene (Figure 1B).

### p-ERK and p-Akt expression in hypergravity-exposed hearts

The levels of phosphorylated ERK (p-ERK) and phosphorylated Akt (p-Akt) increased significantly from 1 to 3 hr post-exposure, but were unchanged immediately after centrifugation. The levels of p-ERK (*P* = 0.007) and p-Akt (*P* = 0.009) in hearts of the 1 hr-interval group were 4.3- and 3.6-fold the levels in the control group, respectively. Moreover, the highest cardiac p-ERK and p-Akt expression was observed 3 hr post-exposure. The levels of cardiac p-ERK (*P* = 0.002) and p-Akt (*P* = 0.004) in the 3 hr-interval group were 6.8- and 5.7-fold the levels in the control group, respectively. After 6 hr post-exposure, cardiac p-ERK and p-Akt levels were not significantly different from those of the control group (Figure 1C).

### eNOS expression in hypergravity-exposed hearts

**Figure 2:**
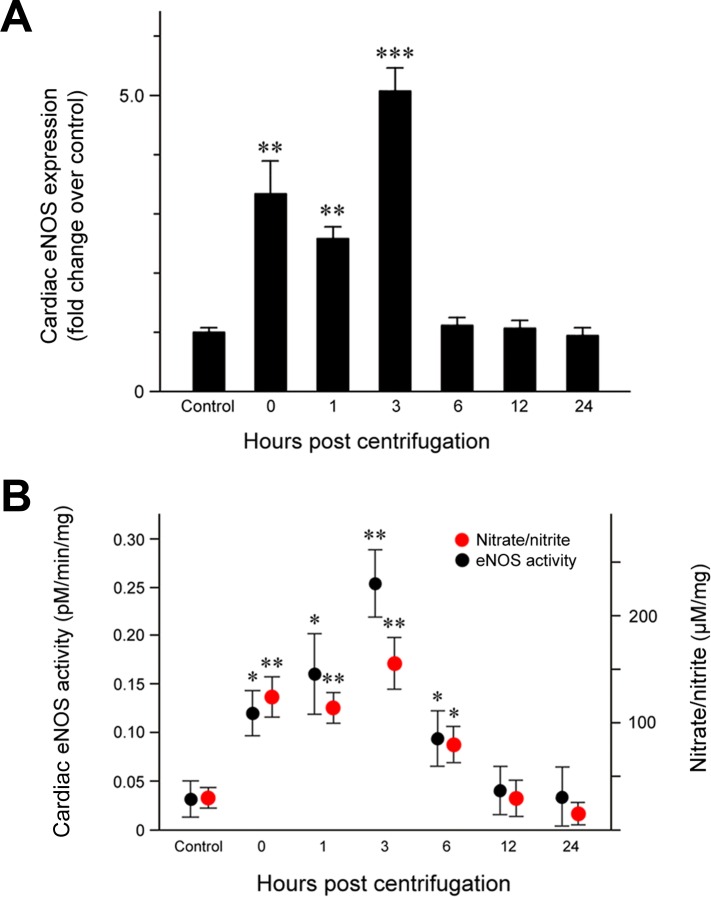
Effect of exposure to hypergravity on cardiac eNOS protein expression and enzymatic activity and NO production **A.** Significantly elevated eNOS protein levels were observed from 1 to 3 hr post-exposure, with two peaks noted between 0 and 3 hr post-exposure. **B.** An L- [^14^C]-citrulline assay and a nitrate/nitrite assay showed similar trends in the levels of eNOS enzymatic activity and nitrate/nitrite concentration, both of which significantly increased from 0 hr to 6 hr post-exposure. Both parameters reached a peak at 3 hr post-exposure. *P < 0.05; **P < 0.01; ***P < 0.001.

Quantitative analysis of eNOS protein expression (Figure 2A) revealed a significant increase immediately after the end of centrifugation (*P* = 0.006), which persisted until 3 hr post-exposure. The highest eNOS level was observed at 3 hr post-exposure (*P* < 0.001) and resulted in a double peak expression pattern. eNOS levels decreased to that of the control group throughout the remainder of the post-exposure period.

### eNOS activity in hypergravity-exposed hearts

We measured the conversion of [^14^C]-labeled L-arginine to L-citrulline in cardiac tissue homogenates to quantify eNOS activity. Our results revealed a concordance between eNOS activity and protein expression: cardiac eNOS activity was significantly elevated immediately after exposure to hypergravity (*P* = 0.026), increased further from 1 (P = 0.019) to 3 hr (*P* = 0.008) post-exposure, and returned to baseline levels at 12 hr post-exposure (Figure 2B).

### Nitrate/nitrite levels in hypergravity-exposed hearts

Cardiac nitrate/nitrite levels showed a trend similar to that of eNOS expression and activity (Figure 2B). Nitrate/nitrite levels in cardiac tissue homogenates were significantly elevated immediately after exposure to hypergravity (*P* = 0.005). After a slightly decrease 1 hr post-exposure, the levels peaked at 3 hr post-exposure (*P* = 0.001), but subsequently decreased to eventually reach values even lower than those of the control group at 24 hr post-exposure.

### Histopathology of hypergravity-exposed hearts

**Figure 3:**
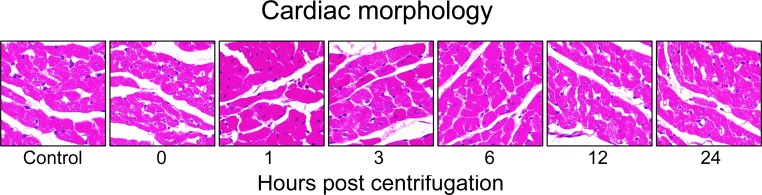
Representative photomicrographs of tissue sections obtained from hypergravity-exposed hearts In all specimens, there was no definitive myocellular necrosis, compensatory hypertrophy or interstitial fibrosis. No significant inflammatory response was detected in the tissue of the myocardium.

Representative photomicrographs are shown in Figure 3. Hearts of the control group displayed no pathologic abnormality. Hearts from the centrifuged mice did not show any significant abnormalities or significant differences in cardiomyocyte size and morphology compared with the control group. In a few areas, the cardiac muscle fibers of hypergravity-exposed hearts appeared separated from each other with foci of mininal myofiber disorganization, and the individual muscle fibers appeared slightly smaller in diameter compared with that of the control group. In all specimens, there was no definitive myocellular necrosis, compensatory hypertrophy or interstitial fibrosis. No significant inflammatory response was detected in the tissue of the myocardium or endocardium, except for occasional isolated neutrophils or lymphocytes, which were extravasated from the blood vessels.

### HIF-1α expression in hypergravity-exposed livers

**Figure 4:**
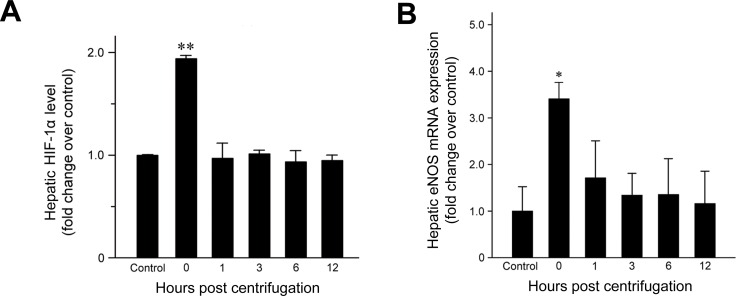
Effects of exposure to hypergravity on hepatic expression of HIF-1α and eNOS mRNA **A.** HIF-1α expression increased significantly in the livers of the centrifuged mice immediately after exposure to hypergravity. This transient rise and rapid decrease were concordant with **B.** significantly elevated eNOS mRNA levels, which were observed at 0 hr post-exposure only. *P < 0.05; **P < 0.01.

HIF-1α expression was examined to confirm that hypergravity induces hypoxia in the livers of centrifuged mice. HIF-1α levels increased significantly immediately after hypergravity exposure (*P* = 0.007; Figure 4A), indicating that hypergravity induces hepatocellular hypoxia. HIF-1α levels decreased to the basal level found in the control group at 1 hr post-exposure and these levels were maintained throughout the remainder of the post-exposure period.

### eNOS expression in hypergravity-exposed livers

Quantitative analysis of eNOS mRNA expression revealed a 3.3-fold increase in the amount of eNOS mRNA at 0 hr post-exposure (Figure 4B; *P* = 0.015). Subsequently, the mRNA levels fell rapidly to basal levels. Throughout the remainder of the post-exposure period, eNOS mRNA levels were maintained at those found in the control group.

**Figure 5:**
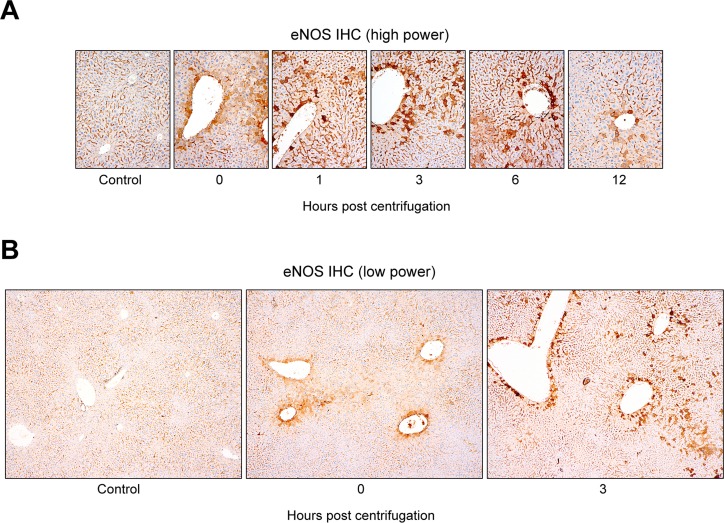
Distinctive hepatocellular eNOS immunostaining pattern induced by exposure to hypergravity **A.** Alteration in hepatic eNOS immunostaining intensity and proportion following exposure to hypergravity. The control group showed no cytoplasmic eNOS immunoreactivity in hepatocytes, except a clear demarcation of bile canaliculi by eNOS. In contrast, the 0-hr interval group showed weak-to-moderate eNOS immunoreactivity in the cytoplasm of some pericentral hepatocytes. In the 1-hr interval group, the number of eNOS-positive hepatocytes with moderate to strong intensity increased significantly. The 3- and 6-hr interval groups revealed a significantly higher proportion of eNOS expression than the 0- or 1-hr interval groups. Furthermore, most cells exhibited strong eNOS expression intensity. The 12-hr interval group showed weak cytoplasmic eNOS immunoreactivity in a few pericentral hepatocytes. **B.** Unique distribution of hepatic eNOS immunoreactivity in hypergravity-exposed mice. No cytoplasmic eNOS expression was observed in the hepatocytes of the control group. The 0-hr interval group exhibited eNOS expression limited to the pericentral (zone 3) hepatocytes around medium-sized vessels. The 3- and 6-hr interval groups displayed strong eNOS expression in the transitional (zone 2) hepatocytes, as well as in the perivascular hepatocytes surrounding large-sized central veins.

Immunohistochemical staining revealed a significant increase in eNOS expression in the livers of centrifuged mice (Figure 5A). The presence of hepatocytes, labeled intensely in brown, in the experimental groups indicated that eNOS expression was upregulated following hypergravity exposure, and this early rise persisted up to 6 hr post-exposure. The control group showed no eNOS expression in the cytoplasm of the hepatocytes, although there was a sharp demarcation of bile canaliculi by eNOS. In contrast, mice exposed to hypergravity showed cytoplasmic eNOS immunoreactivity, although the intensity and proportion were uneven. The 0-hr interval group showed weak-to-moderate eNOS immunoreactivity in the cytoplasm of some pericentral (zone 3) hepatocytes. In the 1-hr interval group, a greater number of eNOS-positive hepatocytes showed high staining intensity than that in the 0-hr interval group. In the 3- and 6-hr interval groups, the number of eNOS-positive hepatocytes clearly increased, and most cells exhibited high staining intensity. The 12-hr interval group showed a decreased number of eNOS-positive hepatocytes, with faint-to-weak staining intensity. In addition, there was a unique distribution of eNOS-positive hepatocytes according to the time course (Figure 5B). The control group showed no staining within hepatocytes. In contrast, the representative eNOS staining patterns in the 0-hr interval group revealed mild eNOS expression, limited to the pericentral hepatocytes around medium-sized vessels. The 3- and 6-hr interval groups displayed strong eNOS expression in transitional (zone 2) hepatocytes and in perivascular hepatocytes surrounding large-sized central veins.

### eNOS activity in hypergravity-exposed livers

**Figure 6:**
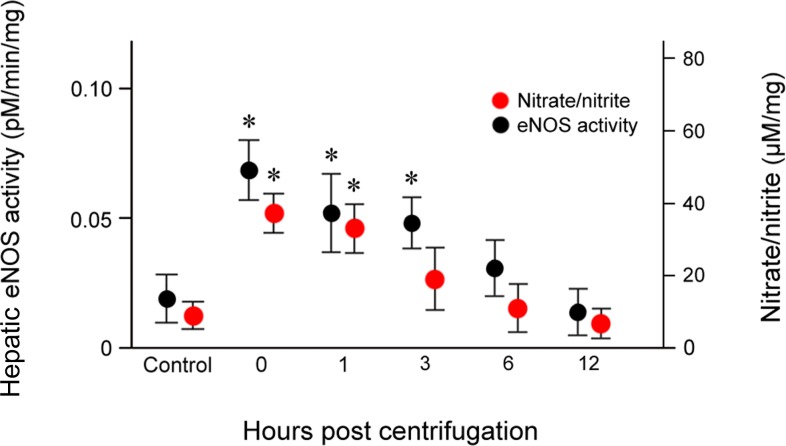
Effects of exposure to hypergravity on the expression of eNOS enzymatic activity of and NO production L- [^14^C]-citrulline and nitrate/nitrite assays showed similar trends in the levels of eNOS enzymatic activity and nitrate/nitrite concentration, both of which significantly increased from 0 hr to 1 hr (eNOS activity) or 3 hr (nitrate/nitrite level) post-exposure. Both parameters peaked 0 hr post-exposure. Results are mean values of three independent experiments in each animal. This increase persisted for 24 hr post-exposure. *P < 0.05.

To quantify hepatic eNOS activity, we measured the conversion of [^14^C]-labeled L-arginine to L-citrulline in liver tissue homogenates. The results revealed concordance between eNOS activity and protein expression. Hepatic eNOS activity increased significantly immediately after exposure (*P* = 0.019), was maintained at significantly elevated levels from 1 (*P* = 0.026) to 3 hr (*P* = 0.028) post-exposure, and returned to baseline levels at 12 hr post-exposure (Figure 6).

### Nitrate/nitrite levels in hypergravity-exposed livers

Hepatic nitrate/nitrite levels showed a trend similar to those of eNOS expression and activity (Figure 6). Nitrate/nitrite levels in liver tissue homogenates increased significantly immediately after exposure to hypergravity (*P* = 0.016). After a slight decrease at 1 hr post-exposure, the levels began decreased further up to 3 hr post-exposure, and continued to fall until they reached values similar to those of the control group at 12 hr post-exposure. None of the animals displayed remarkable behavioral changes during or after centrifugation.

### Histopathology of hypergravity-exposed livers

**Figure 7:**
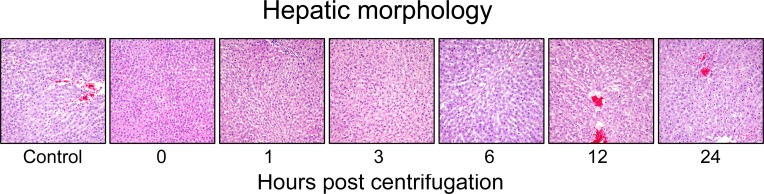
Representative photomicrographs of tissue sections obtained from hypergravity-exposed livers The centrifuged mice showed no significant morphological changes in comparison with those of the control group.

Representative photomicrographs are shown in Figure 7. The centrifuged mice showed no significant alterations in the size, shape or weight of their livers. No significant increase in fibrous connective tissue was observed in the centrifuged livers. There was no significant morphological changes in the centrifuged livers in comparison with those of the control group. The sinusoids were well preserved. No evidence of hepatocellular necrosis, hyperemia, hemorrhage or advanced degenerative change was detected.

## DISCUSSION

In this study, we found that cardiac HIF-1α levels significantly increase immediately after hypergravity exposure, and remain high until 6 hr post-exposure when oxygen tension is normal. A biphasic pattern of HIF-1α induction was observed (i.e., after hypoxia and during reoxygenation). In contrast, HIF-1α mRNA levels were not significantly altered after hypergravity exposure or during the reoxygenation period. These results indicate that cardiac HIF-1α accumulates during both hypoxia and reoxygenation and that its expression is regulated mainly at the protein level.

HIF-1α levels were elevated during reoxygenation as the result of increased protein synthesis and/or stabilization since we did not observe significant changes in HIF-1α mRNA levels. HIF-1α can be detected under normoxic conditions in response to stimulation by various growth factors, hormones, and cytokines [36]. Activation of the PI3K/Akt signaling pathway can lead to increased protein translation and is a major mechanism of normoxic HIF-1α upregulation [37]. In addition, HIF-1α protein synthesis during reoxygenation is known to correlate with activation of the MEK/ERK pathway [38]. Consistent with these data, we found that p-Akt and p-ERK levels increased significantly during the 1 to 3 hr of reoxygenation, whereas their levels were not elevated under hypoxia. The level of p-Akt and p-ERK returned to basal values after 6 hr of reoxygenation. These results are in agreement with previous studies demonstrating that Akt and ERK become transiently activated during the early phase of reoxygenation following hypoxia [1, 39]. Our result for the simultaneous upregulation of HIF-1α, p-Akt, and p-ERK supports the hypothesis that transient activation of ERK and Akt pathways may be involved in increasing cardiac HIF-1α levels during reoxygenation. It is also reasonable to speculate that the Akt and ERK pathways influence HIF-1α stability. Further investigation is required to identify the downstream effectors of Akt and ERK activation in reoxygenation.

In this study, the protein level and activity of eNOS were significantly increased immediately after exposure to hypergravity and during the reoxygenation period. These results were concordant with the time-dependent alteration of cardiac NO production after hypergravity exposure. Although the precise mechanism by which hypergravity causes upregulation of the cardiac eNOS/NO system is unknown, there are several possible explanations. First, previous studies have reported on the subcellular biochemical and ultrastructural alterations in the hearts of experimental animals exposed to hypergravity, indicating that +Gz stress could cause hypoxic myocardial insult [40-42]. Indeed, hypoxia has been associated with eNOS upregulation. Given that the body regularly deals with a wide range of oxygen tensions based on the demands of specific tissues, it is not surprising that the vascular beds of various organs should respond differently to hypoxia, depending on their specific *in vivo* and *in vitro* milieus. Arnet et al. [43] found that hypoxia induces the upregulation of eNOS in the aorta. Justice et al. [22] also demonstrated that hypoxia causes a time-dependent increase in the level of eNOS in coronary microvessels. Xu et al. [23] reported that eNOS regulation is sensitive to oxygen tension and that hypoxia significantly stimulates eNOS activity in coronary arterioles. Moreover, Depre et al. [25] showed that cardiac eNOS is rapidly activated by hypoxia and that the increased eNOS activity is maintained during the entire hypoxic episode. Together with these data, our findings support the notion that hypergravity-induced cardiac hypoxia upregulates eNOS expression. Second, regarding the sustained upregulation of eNOS during the 1-3 hr post-centrifugation period, many studies have indicated a cardioprotective role for NO produced by eNOS in cardiac reoxygenation injury. Brunner et al. [44] and Jones et al. [45] demonstrated that eNOS overexpression increases myocardial tolerance to reoxygenation, as reflected by the improved left ventricular pressure and reduced infarct size. Furthermore, a study using an eNOS-knockout mouse model demonstrated that the loss of eNOS strongly exacerbated myocardial reoxygenation injury following hypoxia [46]. eNOS loss has also been shown to aggravate hypoxia/reoxygenation injury [47]. We speculate that reoxygenation injury might occur when the blood supply returns to the heart after a period of hypoxia due to +Gz exposure and that the increased eNOS levels and activity might induce NO production to protect the myocardium against reoxygenation injury. It is well documented that jet fighter pilots are repeatedly exposed to high +Gz. Under such circumstances, blood is pushed in the direction associated with the G force. It is therefore possible that the oxygen supply to heart muscle decreases to a level below that of the demand, subjecting the myocardium to hypoxia/reoxygenation injury. eNOS upregulation may play a protective role in such situations. Further investigations are necessary to elucidate the mechanism of eNOS upregulation following hypergravity exposure.

Previous studies have suggested that the Akt and ERK pathways are involved in eNOS activation. Fulton et al. [48] demonstrated that Akt directly phosphorylates eNOS, thereby enhancing eNOS activity and NO production. These results link signal transduction by Akt to NO release. Similarly, p-Akt has been shown to phosphorylate and activate eNOS [49]. These data suggest that phosphorylation by Akt is critical for eNOS activation. The relationship between eNOS and the ERK pathway is an area of active investigation. eNOS and ERK physically associate in the cytoplasm [50], and ERK appears to promote eNOS activity [51]. Liu et al. [49] reported that PD-098059, an MEK inhibitor and a downstream inactivator of ERK, abolishes eNOS phosphorylation and NO generation, suggesting a crucial role for ERK in the upregulation of the eNOS/NO system. Our observation of the co-expression of eNOS, p-ERK, and p-Akt raises the possibility that eNOS upregulation during reoxygenation may be partly mediated by the activation of the Akt and ERK pathways.

Goldstein et al. [52] reported the effects of hypergravity on cardiac morphology. After exposure to +2Gz for 14 d, the hearts of centrifuged rats showed regional heterogeneous myofibers, nuclear edema, and binucleation and no myocyte necrosis. In contrast, we did not find any histopathological lesions in the hearts of centrifuged mice. Unlike the hypergravity exposure protocol used by Goldstein et al., our protocol used a rather short duration of exposure (1 hr) and a slightly higher G force (+3Gz *vs*. +2Gz). It may also be possible that hypergravity per se is not the direct cause of tissue hypoxia [53]. Instead, the responses to hypergravitational forces may be an attempt to prevent myocardial infarction and stroke due to possible subsequent ischemic stresses. Further investigations are necessary to clarify the precise mechanism by which hypergravity exposure leads to alterations in cardiac morphology.

We report here the first quantification of eNOS expression in the livers of mice exposed to hypergravity. Real-time RT-PCR analysis and immunohistochemical staining revealed that eNOS expression increased significantly immediately after hypergravity exposure. The early rise in hepatic eNOS levels was concordant with HIF-1α upregulation and eNOS activity. These results suggest that exposure to hypergravity induces hepatocellular hypoxia and has a significant effect on eNOS expression and activity in mouse liver. In addition, we observed that the livers of hypergravity-exposed mice exhibited a significant increase in eNOS immunoreactivity. Increased cytoplasmic eNOS expression was observed immediately after cessation of centrifugation and persisted for 6 hr post-exposure, whereas no cytoplasmic eNOS expression was observed in the hepatocytes of the controls. Although the precise mechanism by which exposure to hypergravity leads to upregulation of hepatic eNOS is unknown, there are some possible explanations. The reflex cardiovascular responses to hypergravity include tachycardia and increase in total peripheral vascular resistance resulting from increase in sympathetic vascular tone. Laughlin et al. [34] suggested that the marked reduction in hepatic blood flow during exposure to hypergravity is the result of sympathetic vasoconstriction in visceral vascular beds. Based on these data, we hypothesized that hepatocellular hypoxia due to hypergravity-induced inadequate hepatic blood flow might be attributable to eNOS upregulation. The post-exposure increase in HIF-1α supports our hypothesis. Furthermore, this hypothesis is also supported by a recent study demonstrating that tissue hypoxia upregulates hepatic eNOS in rats exposed to 10% oxygen [54]. Previous studies suggested that hypoxia is associated with upregulation of eNOS mRNA expression [23, 43, 55]. In this study, we observed weak-to-moderate eNOS expression in some pericentral hepatocytes of the 0-hr interval group, followed by stronger and more frequent eNOS immunoreactivity in transitional hepatocytes of the 3- and 6-hr interval groups. These findings suggest that hypergravity-induced hepatocellular alterations began in zone 3 and extended to the surrounding hepatocytes over time. The area most susceptible to hypoxic hepatocellular injury is zone 3 of the hepatic acini because blood flows through this zone last. Pericentral hepatocytes receive the least oxygenation and are the first to be affected during times of hypoxia. Based on the greater susceptibility of this area of the liver to hypoxic injury, the unique distribution of eNOS expression found here supports our hypothesis that hypergravity-induced hepatocellular hypoxia might cause eNOS upregulation.

Additionally, the possibility that the increase in eNOS expression might be caused by hepatic reperfusion injury cannot be excluded. Hypergravity-induced hepatocellular hypoxia seems insufficient to explain the sustained upregulation of eNOS expression up to 3-6 hr post-exposure. We hypothesized that reperfusion injury might occur when the blood supply has returned to the liver after a period of hypoxia due to hypergravity exposure and that this reperfusion injury might induce eNOS upregulation. These hypotheses are supported by previous data showing that reperfusion after ischemia induces a marked increase in eNOS expression in liver tissue [56, 57]. eNOS plays a hepatoprotective role in the post-ischemic liver by inhibiting cytokines and oxidant release by Kupffer cells [58, 59]. Furthermore, a growing body of evidence from experimental studies substantiates the beneficial effects of eNOS in ameliorating hepatic reperfusion injury [60]. Duranski et al. [56] demonstrated that eNOS overexpression significantly attenuates hepatic tissue injury after ischemia followed by reperfusion. Kawachi et al. [61] observed significant enhancement of hepatic tissue injury in eNOS-deficient mice subjected to ischemia-reperfusion compared with wild-type mice. In addition, Theruvath et al. [31] revealed that eNOS-deficient liver grafts exhibit decreased sinusoidal blood flow velocity and exacerbated reperfusion injury (indicated by increases apoptosis and necrosis, disrupted microcirculation, and enhanced graft infiltration by leukocytes) when compared with the transplanted livers of wild-type mice. In conjunction with these data, our findings raise the possibility that eNOS upregulation during hypergravity exposure might be a defense mechanism to protect the liver from further damage due to ischemia-reperfusion. Further investigations are necessary to clarify the precise mechanism by which hypergravity alters hepatic eNOS expression.

In conclusion, this study is the first to describe hypergravity-induced alterations in the expression of cardiac HIF-1α and eNOS. We observed a significant up-regulation in the levels of HIF-1α and eNOS in the hearts of hypergravity-exposed mice immediately after exposure and during the post-exposure reoxygenation period. In addition, we found that p-Akt and p-ERK expression was significantly elevated during the reoxygenation period, but not immediately after hypergravity exposure. Our results raise suggest that upregulation of HIF-1α and eNOS during reoxygenation may be partly mediated by activation of the Akt and ERK pathways. We also observed significant upregulation of eNOS expression and activity in the livers of hypergravity-exposed mice that persisted for 3-6 hr post-exposure. These results suggest that hypergravity exposure has a significant effect on the upregulation of eNOS in mouse liver. However, further investigations are necessary to confirm or disprove our findings and clarify the precise mechanism by which hypergravity alters hepatic eNOS expression.

## MATERIALS AND METHODS

### Experimental animals and hypergravity exposure

ICR mice at 7 weeks of age were purchased from Samtako Bio Korea Co., Ltd. (Osan-si, Gyeonggi-do, Republic of Korea). Throughout the experimental period, animals were fed standard laboratory mouse chow, provided with free access to water, and maintained on a 12-hour light-dark cycle under pathogen-free conditions with temperature and moisture levels controlled at 20°C to 25°C and 40% to 45%, respectively. Mice were exposed to short-term hypergravity at +3Gz for 1 hr using an animal centrifuge. In the centrifuge, mice were placed inside a cylindrical plastic restraint, which allowed +Gz to be delivered along the rostro-caudal axis. After mice were secured, the restraint was placed into the animal centrifuge, and a cage-mounting module was attached at the end of the arm, which allowed for one degree of freedom to ensure that the net +Gz field was perpendicular to the floor of the restraint device. The control group was treated in an environment identical to that of the centrifuged group with the exception of the +3Gz exposure. The behavior of the mice was monitored with a CCD camera throughout the centrifugation experiments. The Institutional Animal Care and Use Committee of the Republic of Korea Air Force Aerospace Medical Center approved all experimental procedures involving the animals.

To investigate the time course of protein and/or mRNA expression, the centrifuged mice were randomly divided into six groups containing nine to 12 animals per group. At 0, 1, 3, 6, 12, and 24 hr post-exposure, mice were sacrificed by cervical dislocation and laparotomized *via* a midline incision. Heart and liver tissues were immediately removed, preserved in a 10% formaldehyde solution, snap-frozen in liquid nitrogen, and stored at −70°C until further analysis.

### Western blotting

Tissue samples were homogenized in lysis buffer (50 mM Tris, pH 7.5, 1% Nonidet P-40, 2 mM ethylenediaminetetraacetic acid, 10 mM sodium chloride, 20 μg/mL aprotinin, 20 μg/mL leupeptin and 1 mM phenylmethylsulfonyl fluoride) at a volume of 1 mL per 100 mg tissue, and placed on ice for 20 min. After centrifugation at 13000 rpm for 20 min, the supernatant was collected and used for immunoblotting. Immunoblots were incubated with anti-HIF-1α antibody (1:500; Novus Biologicals, LLC, Littleton, CO, USA) or anti-GAPDH antibody (1:800; Novus Biologicals, LLC) followed by incubation with horseradish peroxidase-conjugated secondary antibody (Cell Signaling Technology, Beverly, MA, USA). Protein bands were visualized using an enhanced chemiluminescence reagent (Amersham Biosciences, Buckinghamshire, UK) according to the manufacturer's protocol.

### Quantitative real-time reverse transcriptase-polymerase chain reaction analysis

Total RNA was isolated using the NucleoSpin RNA II extraction kit (Macherey-Nagel GmbH & Co. KG, Dueren, Germany) and used for cDNA synthesis with a ReverTra Ace-α- reverse transcriptase kit (Toyobo Co., Ltd., Osaka, Japan); both kits were used according to the manufacturers' instructions. The amount of standard cDNA was determined photometrically. The cDNA was used for real-time reverse transcriptase-polymerase chain reaction (RT-PCR) using SsoAdvanced SYBR Green Supermix (Bio-Rad Laboratories, Inc., Hercules, CA, USA). PCR was performed using the Bio-Rad CFX96 Real-Time PCR Detection System (Bio-Rad Laboratories, Inc.) with a C1000 Thermal Cycler (Bio-Rad Laboratories, Inc.). PCR reactions for HIF-1α, eNOS, and GAPDH were initiated with a denaturing step at 95°C for 3 min, followed by 40 cycles at 95°C for 10 sec, 58°C for 10 sec, and 72°C for 20 sec. A melting curve (ramping from 65°C to 95°C) was performed following RT-PCR to test for the presence of primer dimers. When primer dimer formation was detected, the PCR was repeated using a separate cDNA aliquot. Each measurement was repeated three times, and the values were used to calculate the ratio of HIF-1α to GAPDH, with the control set at a value of 1.0 to serve as a standard.

### Enzyme-linked immunosorbent assay

Lysates were prepared from tissue samples as described above. The protein concentrations of HIF-1α, p-Akt, p-ERK, and eNOS were determined using commercially available enzyme-linked immunosorbent assay (ELISA) kits (R&D Systems, Inc., Minneapolis, MN, USA).

### Histopathology

Hearts and livers from the control and treated animals were immediately preserved in a 10% formaldehyde solution. After 48 to 72 hours of formalin fixation, the tissues were dissected and processed for routine hematoxylin and eosin staining to assess general architecture and injury.

### Immunohistochemistry

eNOS protein expression was assessed by immunohistochemical staining using the Bond Polymer Intense Detection System (Vision Biosystems, Mount Waverley, Victoria, Australia) according to the manufacturer's instructions. To summarize, 4-μm sections of formalin-fixed, paraffin-embedded tissue were deparaffinized with Bond Dewax Solution (Vision BioSystems), and antigen retrieval was performed using Bond ER Solution (Vision BioSystems) for 30 min at 100°C. Endogenous peroxidases were quenched by incubating sections in hydrogen peroxide for 5 min. Sections were then incubated for 15 min at ambient temperature with a rabbit polyclonal anti-eNOS antibody (1:100; Abcam, Cambridge, MA, USA). The biotin-free polymeric horseradish peroxidase-linker antibody-conjugate system was used in the Bond-maX automatic slide stainer (Vision BioSystems), and visualization was performed using a 3.3-diaminobenzidine (DAB) solution (1 mM DAB, 50 mM Tris-HCl buffer [pH 7.6], and 0.006% H_2_O_2_). Nuclei were counterstained with hematoxylin. Slides were subsequently dehydrated following a standard procedure and sealed with coverslips. To minimize inter-assay variation, positive and negative control samples were included in each run. The positive control sample was normal liver tissue, while the negative control was prepared by substituting non-immune serum for the primary antibody. No staining was detected.

### L- [^14^C]-Citrulline formation assay to measure NOS activity

Tissue samples were ground to a fine powder in liquid nitrogen and homogenized in a solution containing 10 mM 4-(2-hydroxyethyl)-1-piperazineethanesulfonic acid (pH 7.2), 0.32 M sucrose, 0.1 mM ethylenediaminetetraacetic acid, 1 mM dithiothreitol, and protease inhibitors (10 μg/mL each of leupeptin, pepstatin A, chymostatin, antipain, and soybean trypsin inhibitor, and 100 μg/mL phenylmethylsulfonyl fluoride). The homogenate was centrifuged and the supernatants were incubated at 37°C for 60 min with the following: (a) assay cocktail: 2 μM L- [^14^C]-arginine (General Electric Healthcare, Milwaukee, WI, USA), 18 μM L-arginine, 1 mM L-citrulline, 50 mM L-valine, 1 mM dithiothreitol, 0.1 mM NADPH, 0.1 mM tetrahydrobiopterin, 1 mM magnesium chloride, 0.2 mM calcium chloride, and 50 mM potassium hydrogen phosphate, pH 7.2 (Sigma-Aldrich, St. Louis, MO, USA); (b) cocktail with 1 mM ethylene glycol tetraacetic acid (EGTA); or (c) cocktail with 1 mM EGTA plus 1 mM L-N^G^-monomethyl-L-arginine, monoacetate salt, to differentiate Ca^2+^-dependent eNOS activity from Ca^2+^-independent inducible NOS activity. NOS activity was quantified by measuring l- [^14^C]-citrulline with a liquid-scintillation counter (Wallac 1410, Pharmacia LKB Biotechnology AB, Uppsala, Sweden), following removal of unreacted l- [^14^C]-arginine with Dowex 50W-X8 cation-exchange resin (Sigma-Aldrich).

### Nitrate/nitrite measurement

NO production was indirectly quantified by measuring the amount of nitrate/nitrite in supernatants from heart homogenates using a Nitrate/Nitrite Colorimetric Assay Kit (Cayman Chemicals, Ann Arbor, MI, USA) following the manufacturer's instructions. Briefly, 5 μL aliquots were injected into a Sievers Nitric Oxide Analyzer (NOA 280i; Sievers Instruments, Boulder, CO, USA) and pelleted by centrifugation with acetic acid as a reductant for nitrite, and vanadium chloride and hydrogen chloride as reductants for nitrate and sodium iodide. The values were normalized for protein concentration as assessed *via* Bradford reagent. Nitrate/nitrite concentration was expressed as μM based on tissue weight.

### Statistical analysis

All values are provided as mean ± standard error. Differences in the normalized mRNA ratio, protein concentration, enzymatic activity, and nitrite/nitrate concentration between the groups were assessed using a Student's *t*-test (SPSS ver. 18.0 software; IBM SPSS Inc., Chicago, IL, USA), and *P* values < 0.05 were considered statistically significant.
